# Neovagina construction and continent cutaneous urinary reservoir using a previous orthotopic ileal neobladder

**DOI:** 10.1590/S1677-5538.IBJU.2018.0005

**Published:** 2018

**Authors:** Cinthia Alcántara Quispe, Roberto Dias Machado, Wesley Justino Magnabosco, Alexandre Cesar Santos, Eliney Ferreira Faria

**Affiliations:** 1 Departamento de Urologia, Hospital do Câncer de Barretos, Barretos, SP, Brasil

**Keywords:** Urinary Bladder Neoplasms, Cystectomy, Urinary Reservoirs, Continent

## Abstract

Standard radical cystectomy (RC) in women involves removal of the distal ureters, bladder, proximal urethra, uterus, ovaries, and adjacent vagina. Furthermore, pelvic organ-preserving RC to treat selected women has become an accepted technique and may confer better postoperative sexual and urinary functions than standard RC, avoiding complications such as incontinence, prolapse, neobladder-vaginal fistula (NVF), and sexual dysfunction, without compromising oncological outcome.

This article reports a different surgical approach: a patient who underwent a cutaneous continent reservoir and neovagina construction using a previous ileal orthotopic neobladder after RC. Patient presented no complications and she has no evidence of recurrent disease and is sexually active, with a satisfactory continent reservoir. This case is the first report of this procedure that was able to treat concomitant dyspareunia caused by short vagina and neobladder-vaginal fistula.

In conclusion, standard radical cystectomy with no vaginal preservation can have a negative impact on quality of life. In the present case, we successfully treated neobladder fistula and short vagina by transforming a previous ileal orthotopic neobladder into two parts: a continent reservoir and a neovagina. However, to establish the best approach in such patients, more cases with long-term follow-up are needed.

## INTRODUCTION

Bladder cancer is a common disease in women, with ~ 100.000 newly diagnosed cases each year and > 35.000 deaths per year worldwide (1, 2). About 25% of cases comprise muscle-invasive bladder cancer and are treated using RC with pelvic lymphadenectomy, which is the “gold standard” management in cases of high-risk urothelial carcinoma of the bladder. Standard RC in women involves removal of the distal ureters, bladder, proximal urethra, uterus, ovaries, and adjacent vagina. Furthermore, pelvic organ-preserving RC (POPRC) to treat carefully selected women has become an accepted modern technique and may confer better postoperative sexual and urinary functions than standard RC, without compromising oncological outcome (2). Interest in patient's health-related quality of life has promoted the trend towards pelvic organ-preserving techniques (2, 3).

Several complications of neobladder diversion have been described, including incontinence or urinary retention, pouch calculus, prolapse, neobladder-vaginal fistula (NVF) formation, and sexual dysfunction (4, 5). Sexual problems are prevalent after standard RC, especially in younger patients (2, 3), and quality of life and psychological well-being are markedly reduced in women who lack a normal functioning vagina (6, 7).

In the literature, there is no consensus regarding the best surgical method to create a neovagina (6). However, the use of intestinal tissue may even favor good vaginal sexual function: the intestine provides sufficient tissue for the required vaginal depth, it is self-lubricating, and it resembles the vaginal lining in texture and appearance (7-9).

However, to our knowledge, no cases of neovagina construction using the intestine have been published. The present article reports a different surgical approach: the management and outcomes in a patient who underwent cutaneous continent reservoir and neovagina construction using a previous ileal orthotopic neobladder after RC at our institution.

## DESCRIPTION OF CASE

A 37-year-old woman who was married, sexually active, and without comorbidities was submitted for standard RC and extended pelvic lymphadenectomy due to pT2 high-grade urothelial bladder carcinoma (7 cm in diameter) after neoadjuvant chemotherapy (four cycles of “M-VAC”: methotrexate, vinblastine, doxorubicin, and cisplatin). An ileal orthotopic neo-bladder was constructed to allow urinary diversion. Pathological examination of the specimen confirmed high-grade MIBC (pT2N0M0) with negative surgical margins.

**Figure 1:**
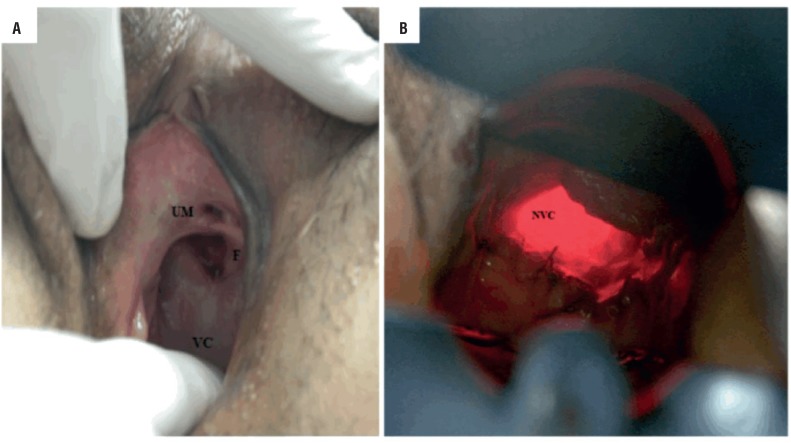
A) Preoperative appearance: Vaginal cul-de-sac (VC), neobladder-vaginal fistula (F), and urethral meatus (UM). B) Postoperative speculoscopy of the neovaginal cavity (NVC).

After 6 months of follow-up, the patient presented with a vaginal-neobladder fistula that required 7-8 diapers per day, as well as dyspareunia. The vaginal orifice of the fistula was located at the short vaginal cul-de-sac (4 cm from the vaginal vestibule; Figure-1A) A correction of the fistula using labial fat flap rotation (Martius technique) was attempted, but the fistula relapsed a few weeks later and remained for more than 2 years, at which point the woman was still complaining of dyspareunia that had led to problems in her relationship.

**Figure 2:**
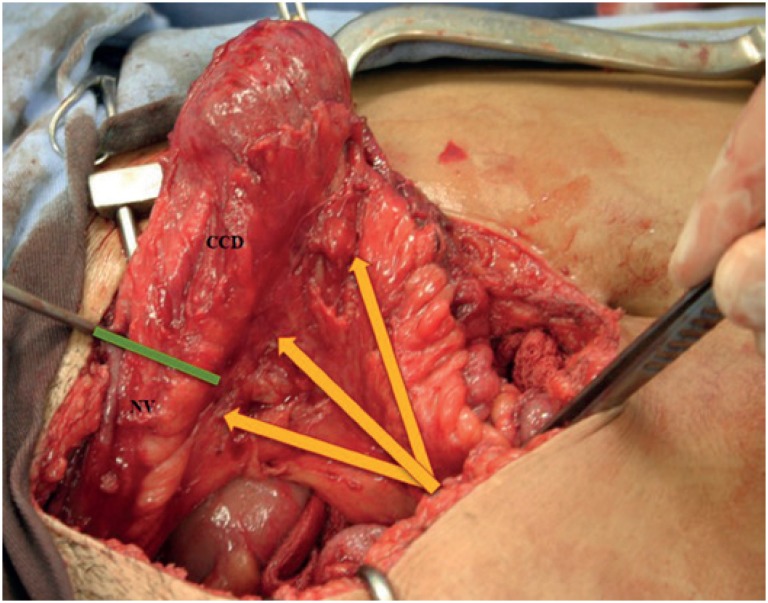
The neobladder is shown exposed and released from its adhesions. The yellow arrows indicate the vascular pedicles, which were used to identify the level of division (green line) between the future neovagina (NV) and the continent cutaneous urinary diversion (CCD).

**Figure 3:**
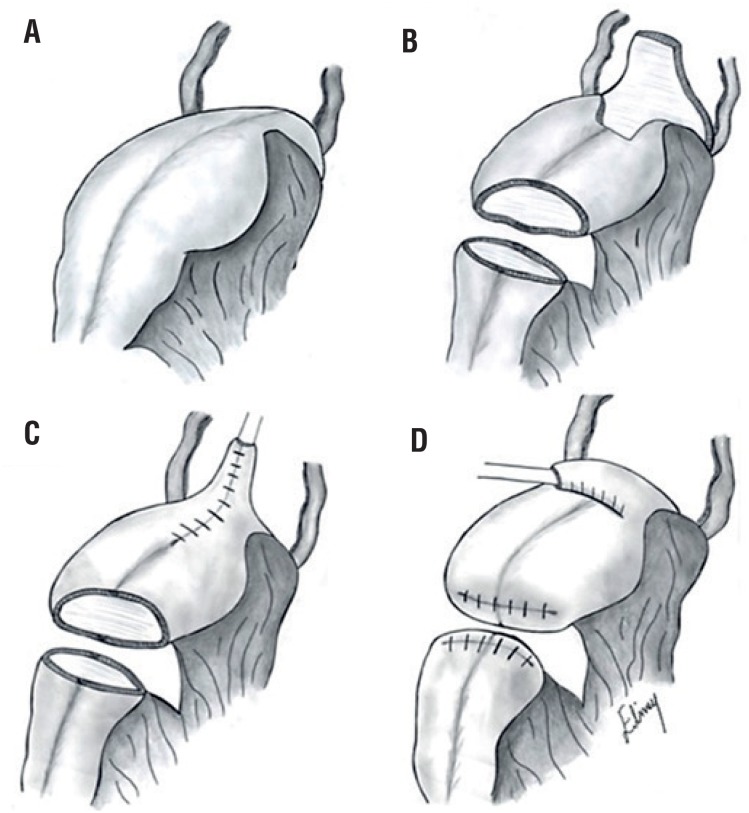
A) Previous neobladder and ureteres. B) Neobladder division and Flap made from the anterior surface of it. C) Creation of the efferent conduit with placed of a urinary catheter. D) Creation of the antireflux valve and closure of distal end of continente cutaneous reservoir. Moreover proximal end of future neovagina it was closed too.

**Figure 4:**
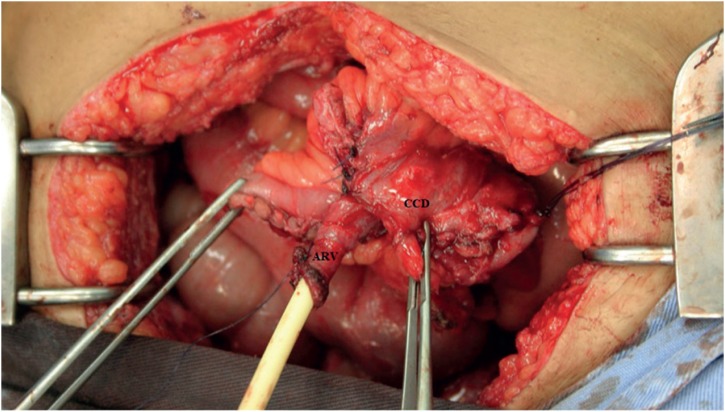
Continent cutaneous urinary diversion (CCD) and anti-reflux valve (ARV).

Surgery was scheduled after the patient had completed a multidisciplinary discussion and signed the consent form. She was submitted to general anesthesia and placed in the dorsal lithotomy position for the duration of the procedure. An infraumbilical laparotomy incision was performed, the neobladder was identified, and the adhesions were released. Using the transillumination technique (Figure-2), the neobladder vascular pedicle was identified. We then divided the neobladder into two parts using a meticulous, vascular-sparing technique: the upper two thirds were used to shape the continent cutaneous urinary diversion, similarly to Macedo's Technique (Figures 3A-D) (10), and the lower one third was used to construct the neovagina. The upper part was detubulized again, and a 3-cm flap was made as an efferent conduit (tube), following Macedo's technique, to create the continence valve mechanism (Figure-4). The distal end of the tube was anastomosed to the skin. A silicone Foley tube was allowed to remain for 4 weeks as an efferent tube. The proximal edge of the lower part was closed, creating a “pocket”, and the perineal time then began. An incision was made that joined the orifice of the fistula to the urethra, augmenting the vaginal cul-de-sac forward to the “pocket” and creating the neovagina (Figure-1B) To prevent prolapse, the neovagina was fixed to the sacral promontory. The final length of the neovagina segment was 15 cm, and a silicon stent was placed 3 weeks after surgery. Antibiotics were given for 7 days after surgery, and long-term dilatation was performed for 6 more weeks until the patient was sexually active. During follow-up, the patient presented no complications, such as vaginal discharge, malodor, vaginal introit stenosis, or dyspareunia, and there were no signs of rupture or abdominal pain related to surgery. At her last appointment after a 10-year follow-up, she had no evidence of recurrent bladder cancer and was sexually active, with a continent reservoir 400 mL in volume.

## DISCUSSION AND FUTURE PERSPECTIVES

Undoubtedly, the ultimate goal of bladder carcinoma treatment and RC is cure of the disease. However, it is also important to provide patients with the best possible functional outcome, because the procedure has an obvious impact on quality of life. In a standard RC, the vaginal vault is incised, and a portion of the anterior vaginal wall (AVW) may even be removed with the specimen. However, a less radical technique has been described-POPRC, which preserves the neurovascular bundle, vagina, and / or uterus. Numerous publications have reported the perioperative and oncologic outcomes of vaginal-sparing radical cystectomy (11), showing potential improvements in quality of life and sexual and urinary function after POPRC. However, the evidence level was low in all of these studies. Furthermore, even if such benefits can be obtained, it is important that oncological outcomes not be compromised (2). Others benefits of POPRC have also been described, such as decreased fistula rate and improved continence (11). NVF is an uncommon but devastating complication that occurs in 3.6% – 10% of women who have undergone RC (4, 12, 13). Its predisposing factors are poor tissue vascularity and damage in the AVW, proximity of suture lines, pelvic radiation, local recurrence, bulking agents used in incontinent patients (4, 5, 14), and local cancer recurrence (13, 14). Several technical modifications have been described that can be used to treat NVF: preservation of the AVW, double vaginal cuff closure, interposition of a well-vascularized omentum flap between the neobladder and vagina, avoidance of overlapping suture lines, and preservation of the clitoral vasculature (2, 11).

Unfortunately, not all patients are amenable to a vaginal sparing approach. Specifically, it is crucial that such a procedure be avoided when the palpable tumor involves the vaginal wall (11), and when no natural surgical path can be negotiated between the posterior bladder and anterior vagina (15).

Quality of life and psychological wellbeing are affected in women who do not have a physiologically normal vagina (6, 7). Intestinal vaginoplasty has become an accepted part of modern vagina reconstruction techniques because it provides enough tissue for the required vaginal depth, self-lubrication, appropriate texture, and an appearance similar to the vaginal mucosa. Furthermore, there is little need for dilatation as there is only a slight tendency to shrink (7, 9). Many women are interested in preserving a good quality sexual life despite having undergone major pelvic surgery (10). In fact, this is one of the main sources of self-reported distress among patients undergoing RC.

No consensus exists regarding the optimal approach or technique to repair naive or recurrent NVF (5, 13). In the present case, we innovated an ileal-based reservoir technique that used part of a previous neobladder with a continent, catheterizable stoma (10). Even though it was a unique case, the procedure was performed successfully, and we were even able to treat concomitant dyspareunia caused by short vagina and neobladder-vaginal fistula.

## CONCLUSION

Standard radical cystectomy with no vaginal preservation may lead to worse postoperative sexual and urinary functions and has negative impact in quality of life. In the present case, we successfully treated neobladder fistula and short vagina by transforming a previous ileal orthotopic neobladder into two parts: a continent reservoir and a neovagina. However, to establish the best approach in such patients, more cases with longterm follow-up are needed.
